# Evaluation of Effect of Ninjin'yoeito on Regional Brain Glucose Metabolism by ^18^F-FDG Autoradiography With Insulin Loading in Aged Mice

**DOI:** 10.3389/fnut.2021.657663

**Published:** 2021-05-12

**Authors:** Jingmin Zhao, Ryota Imai, Naoyuki Ukon, Saki Shimoyama, Chengbo Tan, Yuko Maejima, Yuji Omiya, Kazuhiro Takahashi, Guangxian Nan, Songji Zhao, Hiroshi Ito, Kenju Shimomura

**Affiliations:** ^1^Department of Neurology, China-Japan Union Hospital of Jilin University, Changchun, China; ^2^Department of Radiology and Nuclear Medicine, Fukushima Medical University, Fukushima, Japan; ^3^Tsumura Kampo Research Laboratories, Kampo Research and Development Division, Tsumura & Co., Ibaraki, Japan; ^4^Department of Bioregulation and Pharmacological Medicine, Fukushima Medical University, Fukushima, Japan; ^5^Advanced Clinical Research Center, Fukushima Global Medical Science Center, Fukushima Medical University, Fukushima, Japan; ^6^Department of Neurosurgery, Shanghai East Hospital, Tongji University School of Medicine, Shanghai, China; ^7^Department of Pathophysiology, Basic Medical College of Jilin University, Changchun, China

**Keywords:** Ninjin'yoeito, glucose metabolism, 18F-FDG autoradiography, age, insulin loading

## Abstract

**Introduction:** A recent clinical study revealed that Ninjin'yoeito (NYT) may potentially improve cognitive outcome. However, the mechanism by which NYT exerts its effect on elderly patients remains unclear. The aim of this study is to evaluate the effect of Ninjin'yoeito on regional brain glucose metabolism by ^18^F-FDG autoradiography with insulin loading in aged wild-type mice.

**Materials and Methods:** After 12 weeks of feeding NYT, mice were assigned to the control and insulin-loaded groups and received an intraperitoneal injection of human insulin (2 U/kg body weight) 30 min prior to ^18^F-FDG injection. Ninety minutes after the injection, brain autoradiography was performed.

**Results:** After insulin loading, the ^18^F-FDG accumulation showed negative changes in the cortex, striatum, thalamus, and hippocampus in the control group, whereas positive changes were observed in the NYT-treated group.

**Conclusions:** Ninjin'yoeito may potentially reduce insulin resistance in the brain regions in aged mice, thereby preventing age-related brain diseases.

## Introduction

With a global aging population, health issues caused by aging and age-related diseases have become inevitable challenges to all countries. Understanding the functional changes of organs that occur as a result of aging is essential to prevent age-related diseases, such as Alzheimer's disease (AD) and Parkinson's disease (PD) (1). In particular, the metabolism of glucose as an energy source has been regarded as a potential indicator for these body disorders.

Insulin resistance is one of the major underlying mechanisms in abnormal glucose tolerance (2). Accumulated evidence supports the idea that insulin resistance raises the risk of AD (3) and also correlates with the progression of PD (4). The aged brain has reduced insulin receptor expression levels, diminished insulin transport into the central nervous system, and may even experience insulin resistance (3–7). Therefore, reducing brain insulin resistance is one of the key points to prevent aging-related brain diseases.

Ninjin'yoeito (NYT, Ren-Shen-Yang-Rong-Tang) is a traditional Japanese medicine (Kampo medicine) and a multicomponent formulation composed of 12 crude drug extracts from ginseng, Japanese angelica roots, peony roots, *rehmannia* roots, *Atractylodes* rhizomes, *poria* sclerotium, cinnamon bark, *astragalus* root, *Citrus unshiu* peel, *polygala* roots, *schisandra* fruit, and *Glycyrrhiza*. NYT extract is approved by the Japanese Ministry of Health, Labor and Welfare as a Kampo medicine for the decline in physical strength, fatigue, anorexia, night sweats, cold extremities, and anemia (8). It is also used for individuals with deteriorating physical or psychiatric conditions, particularly among the elderly (9). Ninjin-to is composed of four medicinal herbs, *atractylodes* rhizome, ginseng, glycyrrhiza, and processed ginger, and has been reported to prevent the progression of diabetes mellitus in non-obese diabetic mice (10). Moreover, glucose intolerance in obese mice is alleviated by *astragalus* root through improvement of insulin resistance (11). Interestingly, NYT contains atractylodes rhizome, ginseng, and glycyrrhiza, which are components of Ninjin-to preventing the progression of diabetes (10). Furthermore, NYT also contains astragalus root, which improves insulin resistance (11). On the other hand, a recent clinical study revealed that NYT may potentially improve cognitive outcome and AD-related depression in patients with AD (8). Therefore, we hypothesize that NYT may potentially improve insulin resistance in the brain.

The glucose analog [^18^F]-Fluoro-2-deoxy-2-D-glucose (^18^F-FDG), a molecular imaging probe, is widely used in nuclear medicine for evaluating tissue glucose utilization and glucose metabolism (12–14). In this study, we attempted to clarify the effect of Ninjin'yoeito on regional brain glucose metabolism by ^18^F-FDG autoradiography (ARG) with insulin loading in aged mice.

## Materials and Methods

### Radiopharmaceutical and Reagent

#### NYT Extract

NYT is an herbal supplement composed of 12 crude drugs (15). The NYT extract we used was supplied by Tsumura & Co.

#### ^18^F-FDG

^18^F-FDG supplied for clinical PET examinations, and synthesized by standard procedures was obtained from Fukushima Medical University Advanced Clinical Research Center Cyclotron Facility (Fukushima, Japan). The specific activity of ^18^F-FDG was about 300 GBq/mmol.

### Preparation of Animal Models

**Figure 1:**
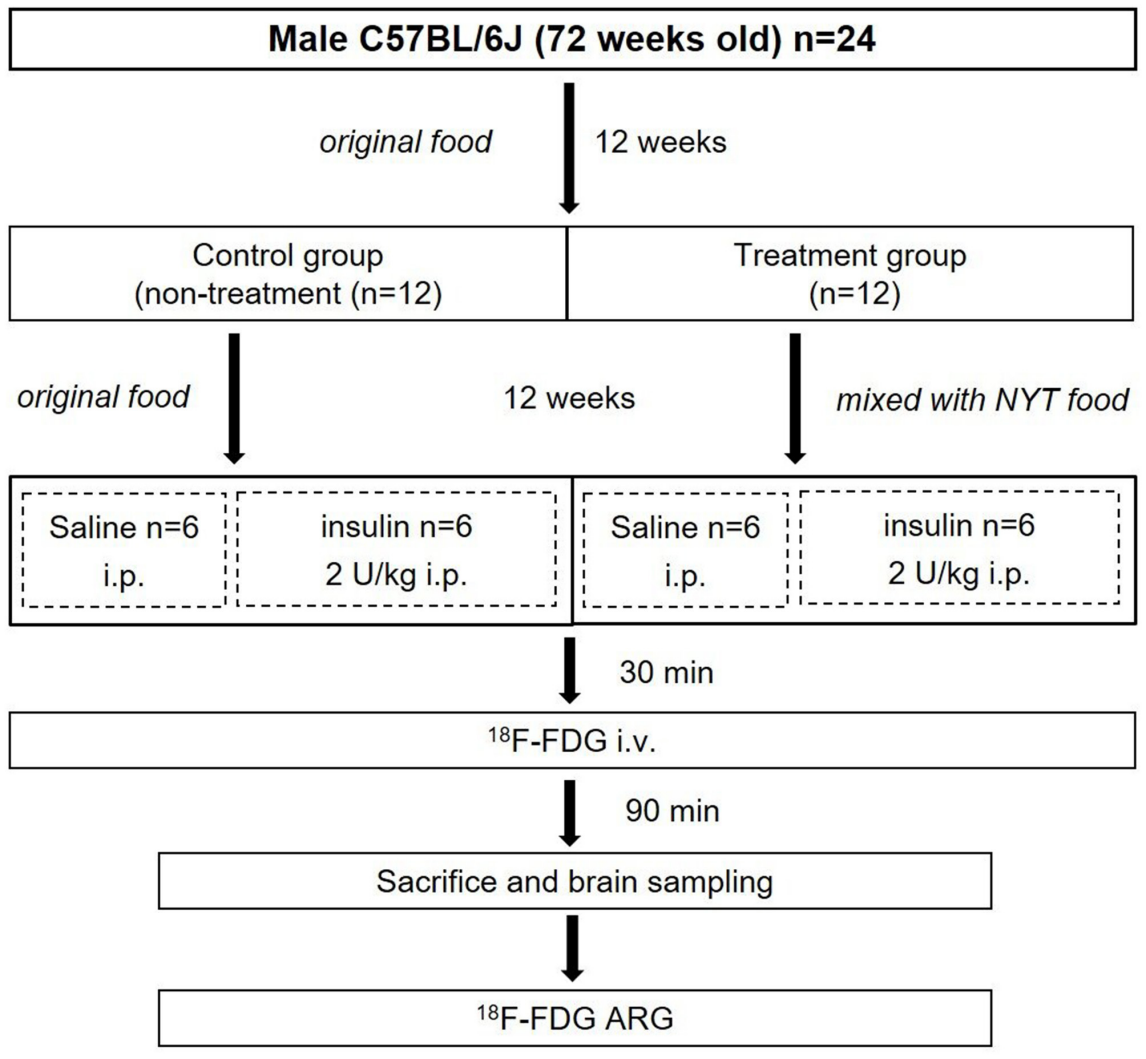
Experimental protocol.

The entire experimental protocol was approved by the Laboratory Animal Care and Use Committee of Fukushima Medical University (Approval Number 30021) and performed in accordance with the Guidelines for Animal Experiments at Fukushima Medical University. Male C57BL/6J mice (72 weeks old) (*n* = 24) were purchased from Charles River Laboratories Japan, Inc. (Yokohama, Japan). All mice were housed in a 12 h light/dark cycle at room temperature maintained at 23–25°C and relative humidity at 45–60%. Food and water were provided *ad libitum*, and the treatment and care of animals met all the criteria of the Association for Assessment and Accreditation of Laboratory Animal Care (AAALAC) International (http://www.aaalac.org/) (16). The experimental protocol is shown in Figure 1. All mice were fed an original diet (CRF-6, ORIENTAL YEAST CO., LTD., Tokyo, Japan). After 12 weeks (84 weeks old), mice were randomly assigned to the control (*n* = 12) and treatment (*n* = 12) groups. The mice in the control group were continued on the original diet (100% CRF-6). The mice in the treatment group were fed the original diet mixed with NYT (97% CRF-6 mixed with 3% NYT, ORIENTAL YEAST CO., LTD., Tokyo, Japan). After 12 weeks (96 weeks old), all mice were fasted overnight and then both the control and treatment groups were further divided two subgroups without and with insulin loading (Figure 1).

### Brain ^18^F-FDG ARG Study

**Figure 2:**
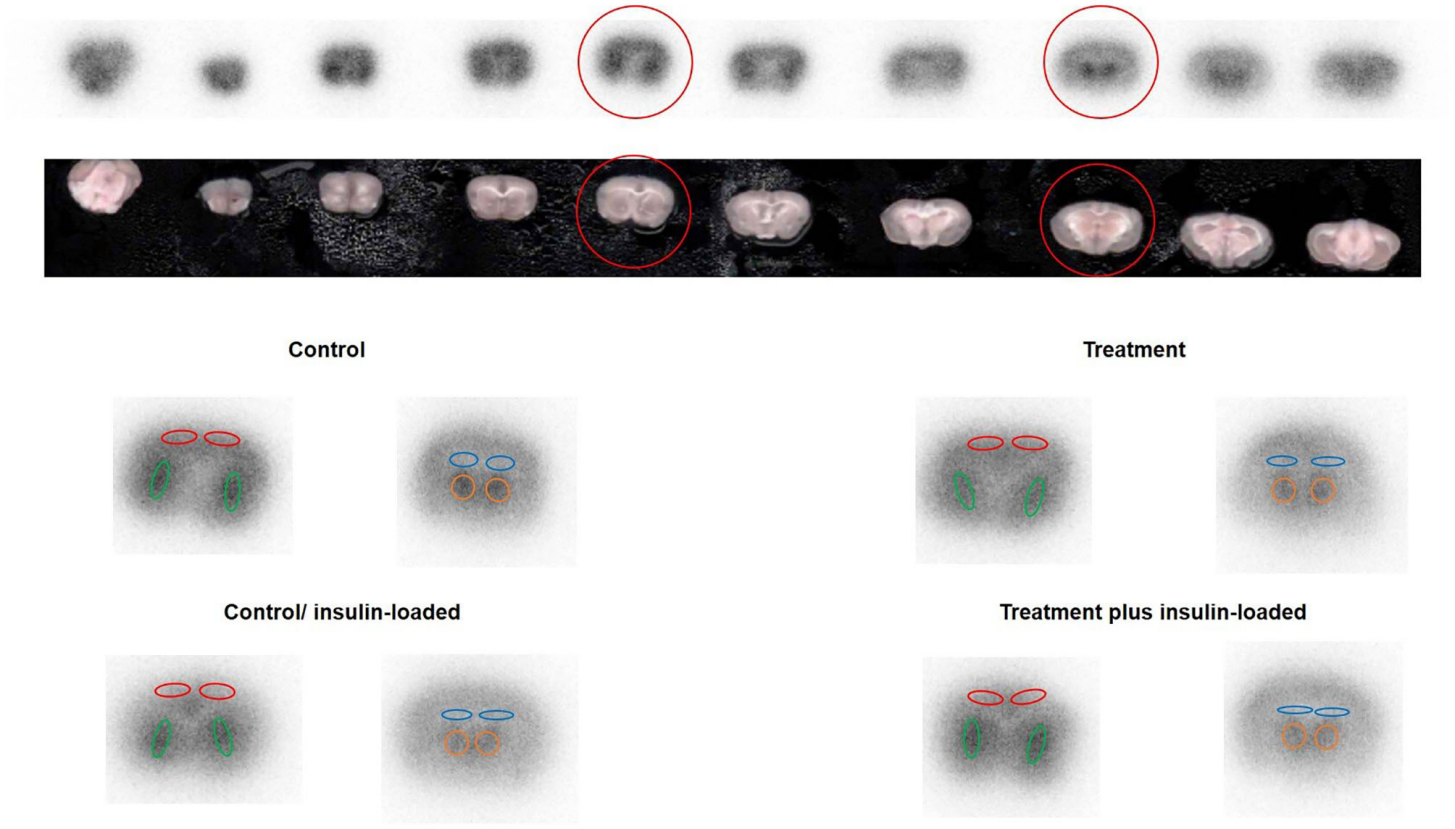
The original picture of brain coronal slices together with the brain ^18^F-FDG ARG image. ROIs were placed on an ^18^F-FDG autoradiography image to cover the cortex, striatum, hippocampus, and thalamus on the left and right hemispheres in four groups. The cortex is encircled in red, the striatum in green, the hippocampus in blue, and the thalamus in orange.

The four subgroups of mice (*n* = 6, each group) were the insulin- and non-insulin-loaded subgroups of the control and treatment groups. The mice in the insulin-loaded subgroups were intraperitoneally injected with human insulin (2 U/kg body weight, Eli Lilly & Co., Kobe) 30 min prior to ^18^F-FDG injection. Each animal was initially anesthetized with 3–4% isoflurane in air and maintained *via* spontaneous ventilation with 2% isoflurane in air. ^18^F-FDG (11.5 MBq/0.1 ml) was injected into the tail vein. Ninety minutes later, the animals were sacrificed; then their brains were rapidly removed, placed in Brain Matrix (Stoelting Co., USA) and cut into coronal slices (2 mm/slice), from which 9–10 coronal slices were obtained for exposure to a phosphor imaging plate (Fuji Imaging Plate BAS-SR 2025 for ^18^F; Fuji Photo Film Co., Ltd., Tokyo, Japan) with a set of calibrated standards (17). This autoradiographic exposure was performed overnight to detect the distribution of ^18^F-FDG. ARG images were analyzed using a computerized imaging analysis system (raytest, CR35, Version 2.1.0, Straubenhardt, Germany) with the image analysis software AIDA (Version 5.1 SP2, Straubenhardt, Germany). To determine brain radioactivity concentration, the cortex, striatum, thalamus, and hippocampus were defined using AIDA. The regions of interest (ROIs), namely, the cortex, striatum, thalamus, and hippocampus in the left and right hemispheres in all mice, were marked on the same anatomical plane with reference to the corresponding brain coronal slices (Figure 2). The radioactivity concentration in each ROI was determined per unit area, and the percentage of injected dose per pixel of the cortex, striatum, thalamus, and hippocampus was obtained and normalized to the animal weight [%ID/pixel/kg body weight (%ID/p/kg)]. Finally, the average of the left and right values around each of the four regions was obtained. The rates of change in ^18^F-FDG accumulation level before and after insulin loading were assessed using the following formula: [(^18^F-FDG uptake level after insulin loading—^18^F-FDG uptake level before insulin loading)/^18^F-FDG uptake level before insulin loading] × 100%. Blood samples for glucose concentration measurement were obtained from all groups. When the blood glucose concentration decreased and displayed as “low” after insulin loading, we defined this blood glucose concentration as 20 mmol/dl which is the detection limit of blood glucose meter.

### Statistical Analyses

All data are expressed as mean ± standard deviation. Statistical analyses were performed using the unpaired Student's *t*-test to evaluate the significance of differences between the control and treatment groups in body weight, blood glucose concentration, and ^18^F-FDG distribution, as well as between non-insulin and insulin-loaded subgroups both in the control and treatment groups. Significance was assumed at *P* < 0.05.

## Results

### Brain ^18^F-FDG Autoradiographic Experiment in Control and Treatment Groups

The body weight and blood glucose concentration were determined in the control and treatment groups (Table 1). The levels of ^18^F-FDG accumulation in brain regions were also determined in the control and treatment groups (Table 2). The body weight and blood glucose concentration were not significantly different between these two groups (Table 1). The levels of ^18^F-FDG accumulation in the cortex, striatum, thalamus, and hippocampus were also not significantly different between these two groups (Table 2).

**Table 1:** Body weight (g) and blood glucose concentration (mg/dl) in brain ^18^F-FDG autoradiography study.

	**Control groups**		**Treatment groups**	
	**Without insulin** **(*n* = 6)**	**With insulin** **(*n* = 6)**	**Without insulin** **(*n* = 6)**	**With insulin** **(*n* = 6)**
Body weight	35.1 ± 4.1	31.6 ± 3.5	32.4 ± 1.8	32.4 ± 3.0
Blood glucose	96.5 ± 9.5	27.2 ± 16.1^****^	90.8 ± 8.0	23.7 ± 6.2^****^

*Data are shown in parentheses (mean ± SD)*.

*Without insulin, no insulin-loaded groups; With insulin, insulin-loaded groups*.

**** *P < 0.0001 vs. value in with insulin-loaded group*.

**Table 2:** ^18^F-FDG accumulation in brain regions in mice (%ID/p/kg).

	**Control groups**		**Treatment groups**	
	**Without insulin** **(*n* = 6)**	**With insulin** **(*n* = 6)**	**Without insulin** **(*n* = 6)**	**With insulin** **(*n* = 6)**
Cortex	0.019 ± 0.002	0.016 ± 0.002^*^	0.017 ± 0.003	0.018 ± 0.002
Striatum	0.024 ± 0.004	0.024 ± 0.004	0.024 ± 0.004	0.028 ± 0.002^#^
Thalamus	0.023 ± 0.002	0.021 ± 0.005	0.021 ± 0.004	0.022 ± 0.003
Hippocampus	0.016 ± 0.002	0.016 ± 0.004	0.014 ± 0.002	0.017 ± 0.003

*Data are shown in parentheses (mean ± SD)*.

*Insulin, insulin-loaded group; Without insulin, no insulin-loaded groups*.

* *P < 0.05, vs. value in without insulin-loaded control group*.

# *P < 0.05, vs. value in with insulin-loaded control group*.

### Changes in ^18^F-FDG Accumulation Level Between Control and Control/Insulin-Loaded Groups

The body weight and blood glucose concentration were determined in the control and control/ insulin-loaded groups (Table 1). The levels of ^18^F-FDG accumulation in brain regions were also determined in the control and control/insulin-loaded groups (Table 2). The body weight was not significantly different between these two groups (Table 1). After insulin loading, the blood glucose concentration significantly decreased (*P* < 0.0001) compared with that in the control group (Table 1). The level of ^18^F-FDG accumulation in the cortex significantly decreased after insulin loading (*P* < 0.05; Table 2).

### Changes in ^18^F-FDG Accumulation Level Between Treatment and Treatment Plus Insulin-Loaded Groups

The body weight and blood glucose concentration were determined in the treatment and treatment plus insulin-loaded groups (Table 1). The levels of ^18^F-FDG accumulation in brain regions were determined in the treatment and treatment plus insulin-loaded groups (Table 2). The body weight was not significantly different between these two groups (Table 1). After insulin loading, the blood glucose concentration significantly decreased compared with that in the treatment group (*P* < 0.0001; Table 1). The levels of ^18^F-FDG accumulation in the striatum and hippocampus tended to increase trend after insulin loading (Table 2).

### Comparison of ^18^F-FDG Accumulation Level Between Control and Treatment Groups After Insulin Loading

The body weight and blood glucose concentration were not significantly different between the control and treatment plus insulin-loaded groups (Table 1). The levels of ^18^F-FDG accumulation in the cortex were higher in the treatment group than in the control group after insulin loading. The levels of ^18^F-FDG accumulation in the striatum were significantly higher in the treatment group than in the control group after insulin loading (*P* < 0.05; Table 2).

### Rates of Change in ^18^F-FDG Accumulation Level Before and After Insulin Loading

**Figure 3:**
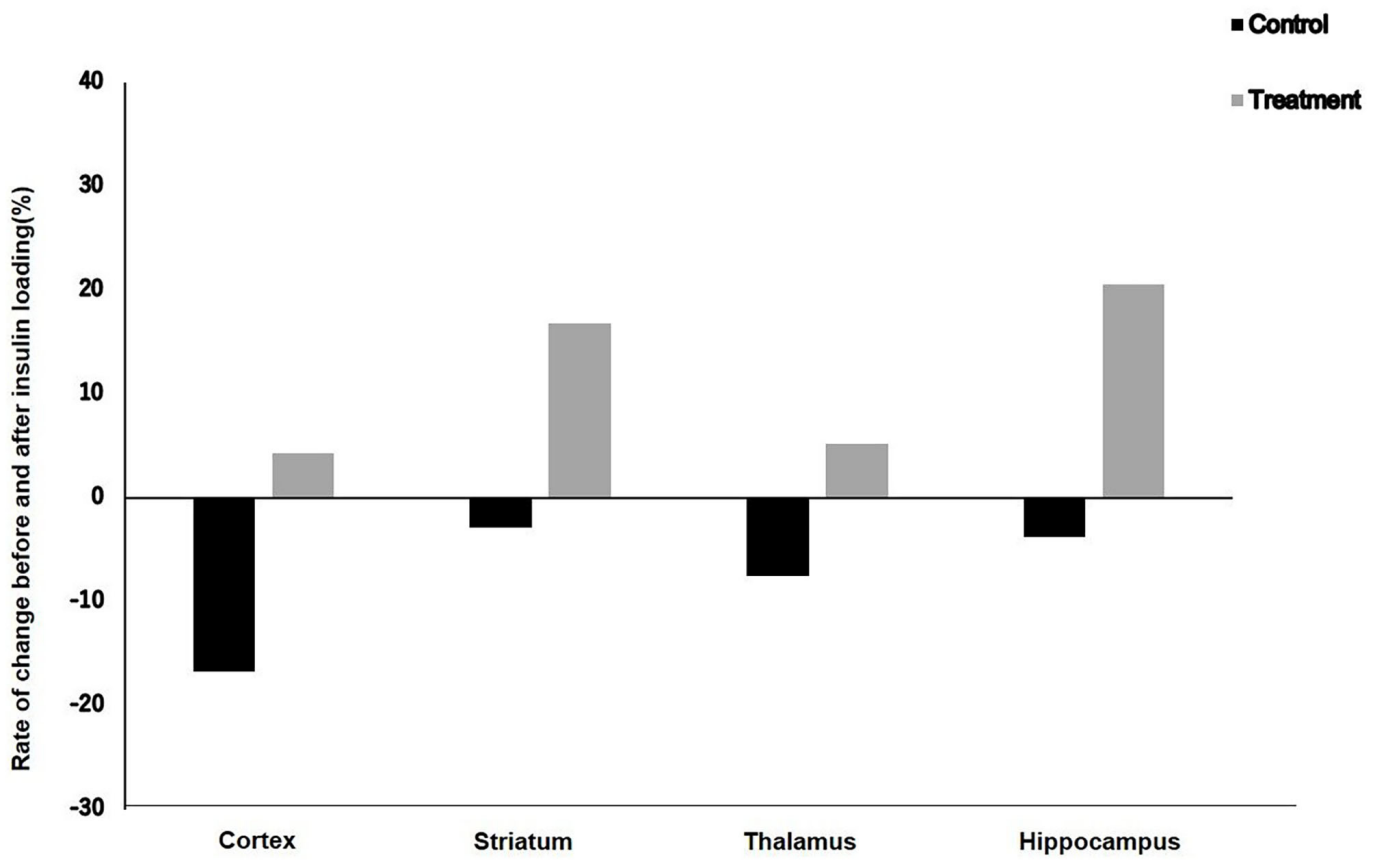
Rate of change in ^18^F-FDG uptake level before and after insulin loading in control and treatment groups.

The rates of change in ^18^F-FDG accumulation level before and after insulin loading were assessed using the following formula: [(^18^F-FDG uptake level after insulin loading—^18^F-FDG uptake level before insulin loading)/^18^F-FDG uptake level before insulin loading] × 100%. The ^18^F-FDG accumulation showed negative changes in the cortex, striatum, thalamus, and hippocampus in the control group, whereas positive changes were observed in the treatment group (Figure 3).

## Discussion

To clarify the effect of Ninjin'yoeito on brain glucose metabolism, we examined and compared the ^18^F-FDG accumulation in brain regions after NYT treatment with insulin loading in aged mice. The ARG method has also been widely used in the quantitative analysis of images of small animal models. ARG imaging plates have a much higher spatial resolution [the Fuji BAS-SR imaging plate is 25–100 μm (17)], than small-animal PET scanners and can visualize the distribution of the radiotracers, within small brain regions and improve the quantification of image analysis of the small brain regions. Therefore, we chose the ARG method to evaluate the effect of NYT on regional brain glucose metabolism in this study.

The highest concentration of insulin receptors was observed in neuronal cell bodies of the hippocampus and cerebral cortex (18, 19). Thalamic atrophy was found to start early in life and has a linear association with age; the same study showed that thalamic atrophy correlated with diminished performance on tests of processing speed (20). Effects of both deficient striatal neurogenesis and age-related neurodegeneration within the striatum accumulate, resulting in a progressive decline in the control functions of the basal ganglia, loss of dopaminergic neurons, and occurrence of PD clinical symptoms (21). On the basis of these studies, we chose the cortex, striatum, thalamus, and hippocampus in the aged brain for analysis in this study.

In this study, the blood glucose concentration and body weight were not significantly different between the control and treatment groups, and between the control and treatment plus insulin-loaded groups. The levels of ^18^F-FDG accumulation in the cortex, striatum, thalamus, and hippocampus were not significantly different between the control and treatment groups. However, the level of ^18^F-FDG accumulation in the cortex was higher in the treatment group than in the control group after insulin loading. Moreover, after insulin loading, the levels of ^18^F-FDG accumulation in the striatum were significantly higher in the treatment group than in the control group. It can be seen from these data that after the insulin loading, the levels of ^18^F-FDG accumulation were higher in the treatment group than in the control group, especially in the cortex and striatum.

On the other hand, in the control group, the level of ^18^F-FDG accumulation in the cortex was significantly decreased after insulin loading, whereas no significant difference found in the treatment group. Insulin as a hormone secreted by pancreatic β-cells, which affects the peripheral system, has been well-characterized. Recent evidence has confirmed that active insulin could also be observed in the central nervous system. Despite the debate about whether insulin is synthesized in the adult brain, it is easily transported across the blood–brain barrier to the central nervous system through a process mediated by saturable receptors (22–24). Elevated peripheral insulin levels will sharply increase the brain and cerebrospinal fluid insulin levels, while prolonged peripheral hyperinsulinemia will downregulate the blood–brain barrier insulin receptors and inhibit the transport of insulin to the brain (25, 26). Insulin receptors are situated on the synapses of both neurons and astrocytes (27). Although insulin and insulin receptors are abundant in the brain, they are selectively distributed, with high concentrations in the cerebral cortex, hypothalamus, hippocampus, olfactory bulb, as well as in the amygdala and septum (23, 28–30). In the literature, it is widely accepted that aging is accompanied by an increase in insulin resistance (31, 32). Bingham et al. (13) demonstrated that glucose metabolism in the cerebral cortex increases significantly after treatment with low-dose insulin. The basis for insulin effects on glucose metabolism in reginal brains may be due to the distribution of glucose transporter isoforms (GLUTs) (33, 34). The insulin-sensitive GLUTs 4 and 8 are selectively distributed in the brain, and insulin enhances brain GLUT 4 expression and translocation (35). In rats, GLUT 4 is expressed in the cerebellum, sensorimotor cortex, hippocampus, pituitary, and hypothalamus (36–39) and GLUT 8 has been observed to be expressed in the hippocampus and hypothalamus (33).

In this study, after insulin loading, the brain ^18^F-FDG uptake level in the cortex of aged mice did not increase but decreased in the control group. This result suggests that there was an obstacle in the cortical glucose metabolism in elderly mice. However, in the treatment group, although the ^18^F-FDG uptake level in the cortex of aged mice did not increase, it did not decrease either. This may be explained by the fact that NYT can improve the insulin resistance of the cortex of aged mice. On the other hand, the ^18^F-FDG uptake levels in the striatum and hippocampus of aged mice did not increase after insulin loading in the control group, whereas an increasing trend after insulin loading was observed in the treatment group. This result suggests that NYT may also improve the insulin resistance in the striatum and hippocampus. After insulin loading, the ^18^F-FDG accumulation showed negative changes in the cortex, striatum, thalamus, and hippocampus in the control group. Previous studies ascribed the reduced ^18^F-FDG uptake levels in tumors and inflammatory lesions with insulin-induced hypoglycemia to the effect of insulin, i.e., insulin shifts ^18^F-FDG from the original area to insulin-sensitive organs (40, 41). This insulin effect may also explain the reduced ^18^F-FDG accumulation level in the control group because of the decreased insulin sensitivity of the brain leading to the shift of ^18^F-FDG to insulin-sensitive organs. However, in the treatment groups, the ^18^F-FDG accumulation showed positive changes in the cortex, striatum, thalamus, and hippocampus after insulin loading. It may indicate that NYT may potentially reduce the insulin resistance in the brain regions of aged mice.

A study showed that insulin resistance in the periphery systems in patients with AD positively correlated with brain amyloid β-protein (Aβ) deposition in the frontal and temporal areas (42). Studies have shown that peripheral insulin resistance may precede the accumulation of Aβ in which midlife Homeostatic Model Assessment for Insulin Resistance (HOMA-IR) predicted Aβ aggregation, as assessed by amyloid positron emission tomography 15 years after HOMA-IR was measured (43). In cognitively healthy adults, compared with Aβ-negative adults, peripheral insulin resistance is also associated with increased levels of Aβ accumulation within 2 years (44, 45). Brain insulin resistance impairs synaptic integrity, and tau and Aβ can also interfere with the actions of insulin at synapses (46). Insulin desensitization may be one of the base of PD disease progression. Clinical data demonstrate that around 8–30% of PD patients are diabetic, a significantly higher percentage than that of the age-matched control group (47–50). Previous studies have documented the importance of insulin signaling in the brain (51–53) and proved that insulin signaling is compromised in the brains of PD patients (54–56). Analogs of incretin hormones have been developed to improve insulin signaling in Type 2 diabetes (57, 58). These drugs enhance insulin release and insulin sensitivity. The antidiabetics in the class of incretin receptor agonists improve symptoms and brain pathology in AD and PD animal models, as well as glucose utilization in AD patients and clinical symptoms in PD patients after their systemic administration (59). The treatment to reduce brain insulin resistance is considered for the treatment of AD and PD.

Hosogi et al. found that NYT improved the serum glucose levels and insulin resistance in STZ-induced diabetic mice (60). This improvement by NYT might be due to the alleviation of interstitial fluid acidification through the increased expression of SMCT1 in the proximal colon leading to the absorption of butyrate, a pH buffer, *via* epithelial cells of the proximal colon (60). The studies reported by Gonçalves and Martel (61) and Gao et al. (62) revealed the mechanism of NYT-induced improvement of insulin resistance: SMCT1 transports butyrate (61), and butyrate intake prevents insulin resistance in high-fat-diet-fed mice (62). These reports (61, 62) suggest that the elevation of SMCT1 expression prevents the occurrence of insulin resistance by increasing the intake of butyrate. The report by Gao et al. (62) indicates that butyrate treatment improves insulin sensitivity by decreasing the levels of blood lipids such as triglycerides, cholesterol, and total fatty acids, which are as critical factors causing insulin resistance. Therefore, the data showing that NYT reduces local brain insulin resistance in aged mice may provide new options for the treatment of AD and PD.

Small-animal PET as a non-invasive, *in vivo* molecular imaging modality has been widely used for preclinical animal models in research facilities, which provide longitudinal investigation of the same subject and voxel-wise analysis. However, most dedicated small-animal PET scanners are limited by their relatively coarse spatial resolution [typically 1 to 2 mm full width at half-maximum (FWHM)] and therefore cannot reliably define specific regions within the mouse brain (63).

The small-animal PET system (Inveon, PET/SPECT/CT) with a spatial resolution of 1.63 mm was installed in our preclinical facility (64). Prior to this study, we have also evaluated the quantitative analysis of ^18^F-FDG PET images of the mouse brain. However, owing to the poor spatial resolution of ^18^F-FDG PET images, it was impossible to accurately define specific regions and set the ROIs at specific regions within the mouse brain. Yang et al. indicated that high-resolution prototype small-animal ^18^F-FDG PET scanner images showed a much higher spatial resolution and a more detailed structure of the mouse brain (63). However, the dedicated small-animal ^18^F-FDG PET scanner (Inveon) could not.

The limitations of our study were as follows. The number of animals per group was small. We only performed image analysis of the coronal sections of the mouse brain, and we did not perform image analysis of the sagittal sections. We consider that more accurate quantitative image information from different sections can be obtained if ARG image analysis of the sagittal sections is performed in this study.

## Conclusion

In summary, Ninjin'yoeito may potentially reduce insulin resistance in the brain regions in aged mice, thereby preventing age-related brain diseases.

## Data Availability Statement

The raw data supporting the conclusions of this article will be made available by the authors, without undue reservation.

## Ethics Statement

The entire experimental protocols were approved by the Laboratory Animal Care and Use Committee of Fukushima Medical University (Approval Number 30021) and performed in accordance with the Guidelines for Animal Experiments at Fukushima Medical University.

## Author Contributions

JZ designed the study and wrote the manuscript. RI, NU, CT, and SS performed animal studies. YO performed bait prescription. KT performed radiolabeling and QC examination. HI and YM contributed to the interpretation of the results. GN, SZ, and KS critically revised the manuscript for important intellectual content. All authors have reviewed the manuscript.

## Conflict of Interest

RI and YO were employed by the company Tsumura & Co. The remaining authors declare that the research was conducted in the absence of any commercial or financial relationships that could be construed as a potential conflict of interest.
